# Genome-Wide Identification and Expression Profiling of the Oxidosqualene Cyclase Gene Family in *Akebia trifoliata* Across Fruit Development and Disease-Susceptibility Groups

**DOI:** 10.3390/cimb48080795

**Published:** 2026-08-06

**Authors:** Hefei Rao, Hao Liu, Jie Li, Xiaoxiao Yi, Yunfeng Deng, Chen Chen, Feiquan Tan, Peigao Luo

**Affiliations:** Key Laboratory of Plant Genetics and Breeding, Sichuan Agricultural University, Chengdu 611130, China; rhfchengdu@163.com (H.R.); lh335837585@163.com (H.L.); lijie@stu.sicau.edu.cn (J.L.); yxx1ao@163.com (X.Y.); huanlu81714@163.com (Y.D.); icbrcc2018@163.com (C.C.); tanfq@sicau.edu.cn (F.T.)

**Keywords:** *Akebia trifoliata*, oxidosqualene cyclase, OSC, triterpenoid biosynthesis, gene family

## Abstract

*Akebia trifoliata* is an important Chinese traditional medicinal plant, and its abundant triterpenoids are the primary active medicinal components. However, the oxidosqualene cyclase (OSC) gene family responsible for constructing their triterpenoid skeletons has not yet been systematically characterized. In this study, nine *AktOSC* genes were identified genome-wide in *A. trifoliata*. Phylogenetic analysis revealed that most AktOSC members formed independent evolutionary subclades. Synteny and selective pressure analyses indicated that multiple duplication modes collectively contributed to the expansion of the AktOSC family, and all members were subjected to strong purifying selection. Sequence alignment showed that all members possess the characteristic motifs of the OSC family, whereas amino acid substitutions at critical active-site residues hinted at potential catalytic product diversity. Cis-acting element prediction revealed an abundance of environmental and phytohormone responsiveness elements within the *AktOSC* promoter regions. Expression profiling revealed that *AktOSC* genes exhibited distinct expression patterns across tissues, developmental stages, and disease-susceptibility groups. Finally, *AktOSC8*, which was specifically and highly expressed in the pericarp, was identified as a highest-priority candidate gene. Collectively, these findings provide essential foundational insights for further elucidating the mechanisms underlying triterpenoid biosynthesis and accumulation in *A. trifoliata*.

## 1. Introduction

Sterols and triterpenoids are important components of plant metabolites with essential roles in membrane stability, cell growth and development, signaling, and responses to biotic and abiotic stresses [1,2,3,4]. Beyond their physiological importance in plants, many triterpenoids also possess substantial pharmacological activities in humans, including anti-inflammatory, anticancer, antiviral, immunomodulatory, and cholesterol-lowering effects, making them valuable resources for the pharmaceutical, nutraceutical, and cosmetic industries [5,6,7].

Oxidosqualene cyclases (OSCs) are the key enzymes that catalyze the cyclization of 2,3-oxidosqualene into a wide variety of sterol and triterpene skeletons [8]. The product outcome of OSCs is closely associated with substrate folding: cyclization in the chair–boat–chair (C-B-C) conformation generally generates a protosteryl cation intermediate and leads to sterol-type tetracyclic products such as cycloartenol and lanosterol, whereas cyclization in the chair–chair–chair (C-C-C) conformation produces a dammarenyl cation intermediate, which typically gives rise to pentacyclic triterpene skeletons such as lupeol and amyrins [9]. The formation of these two conformations is dictated by the unique structural features of the OSC enzyme and the physicochemical properties of key amino acid residues within the catalytic domain [10]. Among these, the DCTAE, QW, and MWCYCR motifs represent the most extensively studied structural elements, and they are essential for catalytic activity and intermediate stabilization, with subtle variations within these regions influencing both substrate folding and the extent of the cyclization cascade [10,11,12]. Because minor amino acid substitutions within the active site can profoundly alter the product outcome [10,13,14], this plasticity has driven the extensive structural variation in triterpene skeletons and prompted the emergence of multiple OSC subfamilies, including cycloartenol synthases (CASs), lanosterol synthases (LASs), lupeol synthases (LUPs), dammarenediol synthases (DDSs), β-amyrin synthases (bASs), and multifunctional OSCs [4,15,16].

*Akebia trifoliata* (Thunb.) Koidz., a perennial woody vine of the family Lardizabalaceae [17], is a traditional medicinal plant whose stems and fruits are both used in traditional Chinese medicine (TCM) as “Mutong” and “Yuzhizi” [18,19], respectively. These two distinct medicinal parts are employed for different therapeutic indications: Mutong is exploited to treat neuralgia, menstrual pain, inflammatory disorders, and infections of the kidneys and urinary tract, whereas Yuzhizi is primarily prescribed for indigestion, inflammation, cardiovascular diseases, and as an adjuvant therapy for malignant tumors [18,19]. This divergence in clinical applications is fundamentally driven by variations in their secondary metabolite profiles. While Mutong accumulates a complex mixture of triterpene saponins, phenylethanoid glycosides, and lignans, Yuzhizi features a more pronounced enrichment of oleanane-type triterpenoids [19]. Recent phytochemical studies have revealed that the triterpenoid aglycones in *A. trifoliata* are predominantly composed of hederagenin, followed by arjunolic acid, dehydroarjunolic acid, oleanolic acid, and dehydrooleanolic acid [20,21]. The attached sugar moieties mainly include glucose, rhamnose, xylose, and arabinose. These triterpene saponins have been demonstrated to possess potent antitumor and anti-inflammatory activities, representing the primary source of medicinal value for both Mutong and Yuzhizi [20,22,23].

From a safety perspective, historical incidents of aristolochic acid-associated nephrotoxicity linked to medicinal materials marketed as Mutong were primarily attributed to the use of *Aristolochia manshuriensis* (“Guanmutong”), rather than *Akebia*-derived materials [18]. LC–UV/LC–MS analyses, including a subsequent multilaboratory validation, found aristolochic acid I to be below the reporting limit (<2.00 μg g^−1^) in the tested stem materials of *A. trifoliata* [24,25]. In a comparative rat study, *Akebia quinata*, another recognized botanical source of Mutong, caused no detectable renal injury at the tested dose, whereas *A. manshuriensis* induced marked nephrotoxicity attributable to its aristolochic acid content [26]. Collectively, these chemical and toxicological findings indicate that *Akebia*-derived medicinal materials carry a substantially lower risk of aristolochic acid-associated nephrotoxicity than *A. manshuriensis*.

Despite decades of extensive phytochemical and pharmacological characterization that has established the medicinal value of *A. trifoliata*, functional genomic research into this species remains in its infancy [27]. As a typical non-model medicinal plant, *A. trifoliata* lacks a stable and efficient genetic transformation system, which imposes a severe bottleneck on its molecular and genetic investigations and renders direct in vivo functional validation of metabolic genes highly challenging [28,29]. Under these methodological constraints, genome-wide bioinformatic analysis emerges as an indispensable research strategy rather than a mere computational tool. By utilizing comparative genomics, molecular evolutionary analysis, and expression profiling, a systematic genome-wide study can effectively circumvent current technical limitations to screen core candidate members within a gene family, thereby providing a critical theoretical foundation for future functional characterization and genetic engineering once transformation platforms mature.

In this study, we performed a genome-wide identification and comprehensive analysis of the OSC gene family in *A. trifoliata*. Specifically, we identified OSC family members from the reference genome, analyzed their gene and protein structures, conserved motifs, and chromosomal distributions, and inferred their evolutionary relationships and duplication history. We also examined cis-acting regulatory elements in the promoter regions and explored their expression patterns using available transcriptomic data, with the aim of prioritizing candidate *AktOSC* genes that may participate in triterpenoid biosynthesis. This work provides a foundation for future functional characterization of *AktOSC* genes and for elucidating the molecular mechanisms underlying triterpenoid metabolism in *A. trifoliata*.

## 2. Materials and Methods

### 2.1. Data Sources

The genome assembly and annotation files of *A. trifoliata* (accession no. GWHBISH00000000) were retrieved from the National Genomics Data Center (NGDC, https://ngdc.cncb.ac.cn/; accessed on 15 March 2024) [30]. Concurrently, the reference genome sequences and corresponding annotation files of *Arabidopsis thaliana* and *Oryza sativa* were downloaded from the Ensembl Plants database (https://plants.ensembl.org/; accessed on 19 March 2024 and 19 November 2024, respectively). The OSC protein sequences of *A. thaliana* (*n* = 13) [16] and *O. sativa* (*n* = 12) [31] were obtained from the TAIR database (https://www.arabidopsis.org/; accessed on 15 January 2026) and the Rice Genome Annotation Project database (https://rice.uga.edu/; accessed on 15 January 2026), respectively. Hidden Markov model (HMM) profiles of the squalene-hopene cyclase C-terminal domain (PF13243) and N-terminal domain (PF13249) were downloaded from the InterPro database (https://www.ebi.ac.uk/interpro/; accessed on 15 January 2026). Additionally, a total of 44 reviewed sequences belonging to the terpene cyclase/mutase family were collected from the UniProt database (https://www.uniprot.org/; accessed on 20 March 2026), spanning 14 species that range from the early-diverging green alga to the core eudicot.

Furthermore, two publicly available transcriptome datasets of *A. trifoliata* were utilized in this study. The fruit growth and development transcriptome data (NCBI BioProject accession no. PRJNA671772; https://www.ncbi.nlm.nih.gov/bioproject/671772; accessed on 30 March 2026) comprised 36 RNA-seq libraries generated from flesh, seed, and peel tissues at four developmental stages (immature, enlargement, coloring, and mature), with three biological replicates for each tissue–stage combination [27]. The disease-associated transcriptome data (NGDC BioProject accession no. PRJCA014987; https://ngdc.cncb.ac.cn/bioproject/browse/PRJCA014987; accessed on 30 March 2026) comprised nine peel RNA-seq libraries representing disease-resistant (RP), disease-susceptible (SP), and control (CP) samples, with each sample category collected during three sampling periods (May, June, and July) [32]. According to the BioProject metadata, these samples were collected under naturally occurring disease conditions without artificial pathogen inoculation. The RP and SP libraries were generated from the mixed pool of 24 lines exhibiting disease resistance and 50 lines exhibiting susceptibility to fungal disease, respectively, whereas the CP libraries were derived from fruit peels of the genome-sequenced cultivar ‘Shusen 1’.

### 2.2. Genome-Wide Identification of A. trifoliata OSC Gene Family

To systematically identify OSC gene family members within the *A. trifoliata* genome, a combination of hidden Markov model and homology-based BLAST searches was employed. Initially, the HMMER software (v3.3.2) was utilized to screen the genome assembly using the Pfam profiles PF13249 and PF13243, with an E-value threshold of 1 × 10^−5^ and default parameters for all other settings [33]. Concurrently, local BLASTP was performed using NCBI BLAST+ (v2.17.0+) against the *A. trifoliata* proteins using the known OSC protein sequences from *A. thaliana* (*n* = 13) and *O. sativa* (*n* = 12) as queries under the identical E-value cutoff [34].

The candidate proteins intersected from both the HMMER and BLASTP pipelines were subsequently submitted to the SMART database (https://smart.embl.de; accessed on 20 January 2026) to verify the presence of the OSC conserved domains [35]. Sequences that lacked a complete conserved domain were manually discarded. The remaining proteins were confirmed as high-confidence *A. trifoliata* OSC family members and designated as AktOSC proteins.

### 2.3. Physicochemical Property and Subcellular Localization Analysis

ProtParam on the ExPASy server (https://web.expasy.org/protparam/; accessed on 22 January 2026) was employed to predict the physicochemical properties of the AktOSC proteins [36]. Additionally, their subcellular localizations were analyzed using DeepLoc 2.1 (https://services.healthtech.dtu.dk/services/DeepLoc-2.1/; accessed on 25 January 2026) [37].

### 2.4. Protein Domain, Conserved Motif, and Gene Structure Characterization

The conserved functional domains of AktOSC proteins were analyzed using the NCBI Batch CD-Search tool (CDD, v3.21; https://www.ncbi.nlm.nih.gov/Structure/bwrpsb/bwrpsb.cgi; accessed on 30 January 2026) [38]. Conserved motifs within the proteins were predicted using the MEME Suite (MEME Suite, v5.5.9; https://meme-suite.org/meme/tools/meme; accessed on 30 January 2026) [39], with the maximum motif width set to 75 and the total number of motifs configured to 8, while maintaining default settings for all other parameters. Concurrently, the exon-intron structures of the *AktOSC* genes were extracted from the GFF3 file. Finally, the identified protein domains, conserved motifs, and gene structures were integrated and visualized using TBtools (v2.476) [40].

### 2.5. Phylogenetic Analysis

The AktOSC protein sequences, along with 44 reference OSC proteins retrieved from diverse plant species, were aligned using MAFFT (v7.525) [41]. The resulting multi-sequence alignment was automatically trimmed using trimAl (v1.5) to remove poorly aligned regions [42]. Subsequently, an ML phylogenetic tree was constructed using IQ-TREE (v2.3.6) [43]. The best-fit amino acid substitution model was automatically selected using the ModelFinder Plus algorithm, and branch support was evaluated with 1000 bootstrap replicates. The resulting tree file was visualized using the Interactive Tree Of Life platform (iTOL, v7; https://itol.embl.de/; accessed on 20 March 2026) [44].

### 2.6. Chromosomal Location, Gene Duplication, Collinearity and Ka/Ks Analysis

The physical chromosomal positions of the *AktOSC* genes were determined based on the *A. trifoliata* genome annotation file and visualized using TBtools. To investigate syntenic relationships, collinearity analyses of the *OSC* genes between *A. trifoliata* and two reference species (*A. thaliana* and *O. sativa*) were performed using the MCScanX [45] module integrated within TBtools, maintaining default parameters. Gene duplication events within the *AktOSC* genes were identified based on the outputs generated by the MCScanX module. Subsequently, the Ka/Ks ratio of the identified *AktOSC* genes was calculated by TBtools.

### 2.7. Analysis of Cis-Acting Elements

The putative cis-acting elements located in the promoter regions (2000 bp upstream of the start codon) of the *AktOSC* genes were computationally identified via the PlantCARE database (https://bioinformatics.psb.ugent.be/webtools/plantcare/html/; accessed on 26 March 2026) [46]. The predicted results were subsequently classified and visualized using TBtools.

### 2.8. Expression Analysis

The two public transcriptome datasets described in Section 2.1 were analyzed separately. RNA-seq reads were mapped to the *A. trifoliata* reference genome using HISAT2 (v2.1.0) [47]. Gene expression levels were quantified as fragments per kilobase of transcript per million mapped fragments (FPKM) using StringTie (v1.3.6) [48]. Subsequently, the FPKM values of the *AktOSC* genes were extracted and transformed as log_2_(FPKM + 1). Finally, expression heatmaps of the *AktOSC* genes were generated using TBtools.

## 3. Results

### 3.1. Identification of OSC Gene Family Members in A. trifoliata

A total of nine high-confidence *AktOSC* genes were systematically identified within the *A. trifoliata* genome (Table 1). Based on their genomic distribution, the nine genes were sequentially designated as *AktOSC1* to *AktOSC9*. Chromosome 8 harbored the highest number of *AktOSC* genes, with three members forming a tandem cluster at the chromosome terminus (Figure S1). Although the genomic lengths of these nine *AktOSC* genes exhibited considerable variation, ranging from 10,570 to 24,638 bp, their coding sequence (CDS) lengths (2121–2310 bp) and the number of exons (17–20) were highly conserved.

Furthermore, the physicochemical properties of the nine putative AktOSC proteins displayed a high degree of similarity, with amino acid lengths of 706–769 amino acids (AA), molecular weights of 80.31–88.32 kDa, theoretical isoelectric points (pI) of 5.86–6.51, aliphatic indices of 76.85–81.94, and grand average of hydropathy (GRAVY) values of −0.372 to −0.269. Notably, with the exception of AktOSC6, which was predicted to be stable with an instability index of 36.60, all other AktOSC proteins were classified as unstable, with their instability indices ranging from 43.66 to 50.13. Subcellular localization analysis showed that all AktOSC proteins were consistently localized to the endoplasmic reticulum (ER).

### 3.2. Gene Structure, Conserved Domain, and Motif Analysis

**Figure 1:**
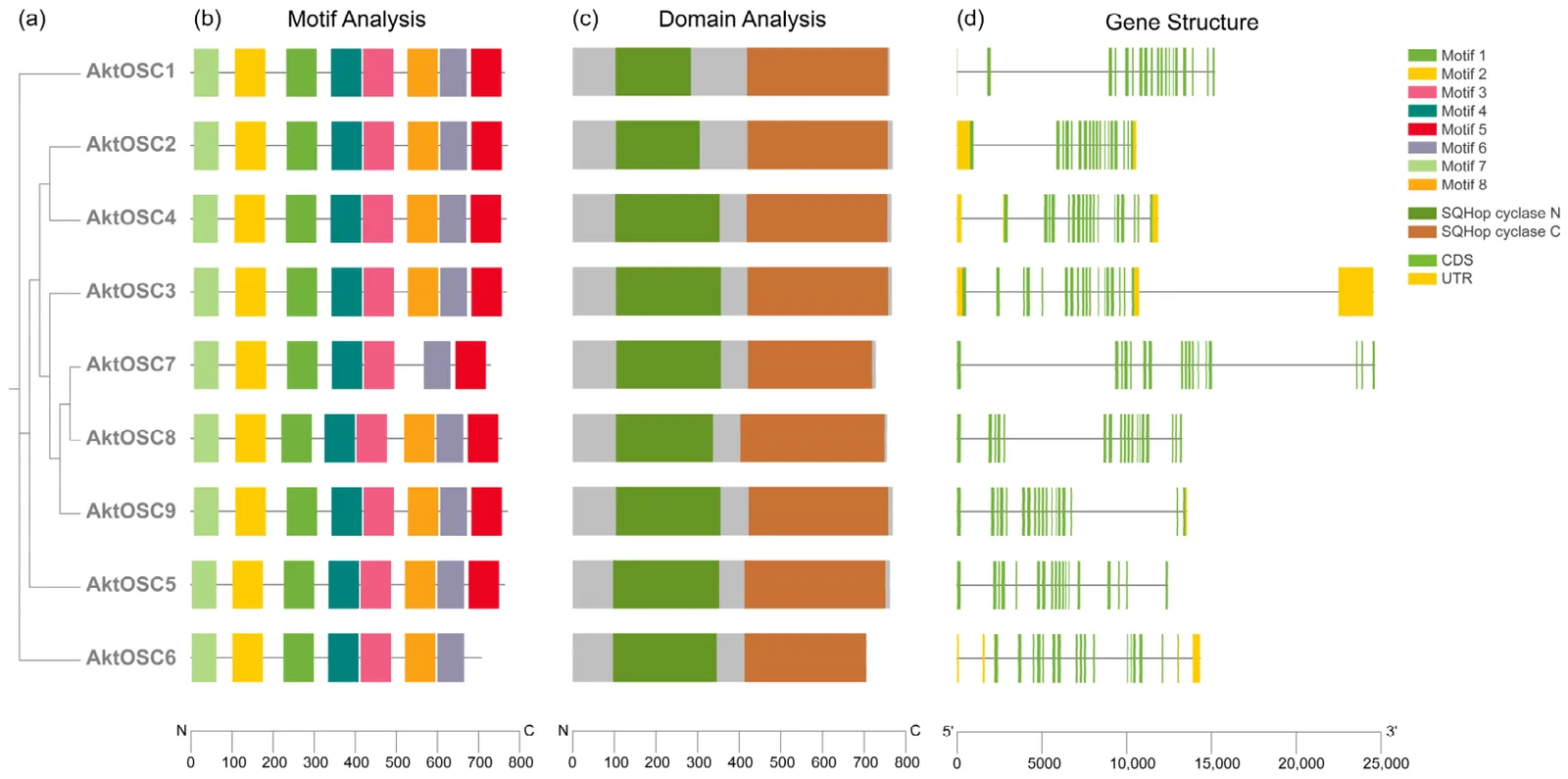
Structural and evolutionary analysis of the *AktOSC* gene family. (**a**) Phylogenetic relationship among the nine AktOSC proteins. (**b**) The distribution of protein conserved motifs. Different colored boxes indicate distinct motifs. (**c**) Conserved domain architecture. Green and brown boxes indicate SQHop_cyclase_C and SQHop_cyclase_N domains, respectively, and grey boxes indicate regions outside the annotated domains. (**d**) Exon-intron structures. Green boxes, yellow boxes, and black lines represent CDS, untranslated regions (UTR), and introns, respectively. Scale bars at the bottom indicate protein length (AA) for (**b**,**c**), and gene length (bp) for (**d**).

To reveal the structural characteristics of the *AktOSC* gene family, their exon–intron organizations and protein motif distributions were systematically characterized. MEME analysis identified eight conserved motifs within the AktOSC proteins, with Motif 5 and Motif 8 being specifically absent in AktOSC6 and AktOSC7 (Figure 1), respectively. Detailed sequence inspection verified that these predicted motifs encompassed the essential catalytic features required for oxidosqualene cyclization (Table 2). Motif 3 harbored the highly conserved DCTAE motif, which is directly associated with the OSC catalytic reaction. Furthermore, Motif 1 contained the MWCYCR signature sequence, which is a documented hallmark of pentacyclic triterpene synthases. Additionally, Motifs 2 and 6 each carried two copies of the conserved QX_2–5_GXW motif, which is important for the stability of OSC catalytic reaction. Concurrently, conserved domain analysis confirmed that the classic dual-domain architecture, comprising the squalene-hopene cyclase N-terminal and C-terminal domains, was intact across all identified AktOSC proteins (Figure 1).

Gene structure analysis indicated that *AktOSC* genes typically exhibited complex architectures, containing 17 to 20 exons (Figure 1). While the genomic lengths of the majority of *AktOSC* genes fell within a relatively uniform range of approximately 10 to 15 kb, *AktOSC3* and *AktOSC7* exhibited larger genomic regions of approximately 24 kb, a phenomenon primarily attributed to the substantial elongation of their intronic regions.

### 3.3. Phylogenetic Analysis of AktOSC Proteins

**Figure 2:**
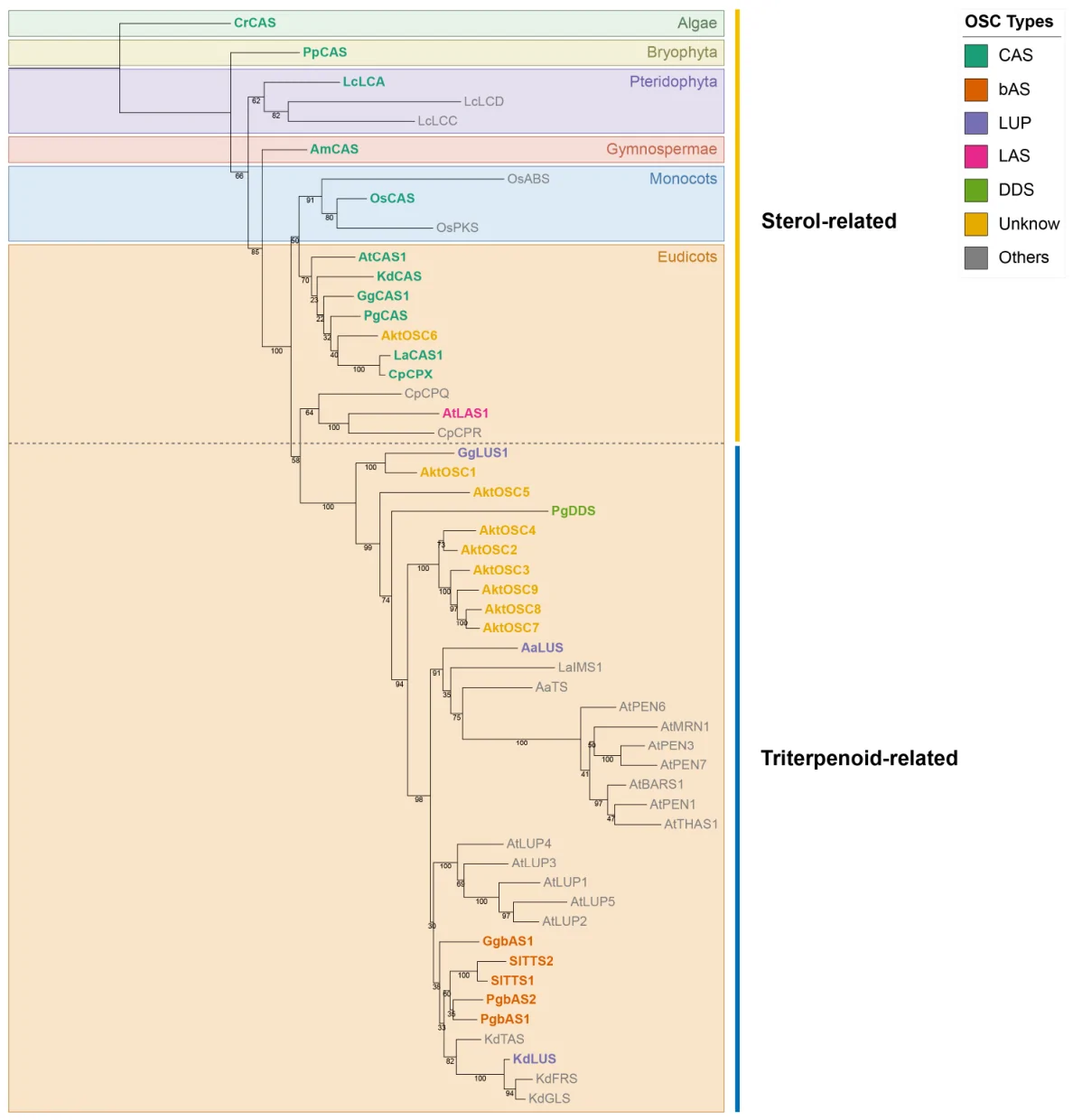
Phylogenetic relationships of nine AktOSC proteins and 44 reference OSC proteins from various plant species. The phylogenetic tree was constructed using the ML method based on amino acid sequences, with bootstrap support values from 1000 replicates indicated at the nodes. The CAS sequence from *Chlamydomonas reinhardtii* was utilized as the outgroup. Functional categories of OSCs are distinguished by distinct label colors, with gray representing multi-product/uncommon types and yellow denoting uncharacterized AktOSCs. Dashed lines demarcate the boundary between the sterol biosynthesis and triterpenoid biosynthesis clades. Detailed information for all analyzed OSCs is provided in Table S1.

To investigate the evolutionary relationships and predict the functional contexts of the AktOSC proteins, a maximum likelihood (ML) phylogenetic tree was constructed using the amino acid sequences of the nine AktOSC proteins alongside 44 functionally characterized reference OSC proteins from diverse plant species (Figure 2, Table S1). The phylogenetic topology resolved into multiple clades and corresponding OSC functional categories, based on which the AktOSC proteins were classified. Specifically, AktOSC6 clustered robustly within the CAS clade with strong bootstrap support, suggesting high confidence in its orthology to known sterol-synthesizing enzymes. Meanwhile, AktOSC1 grouped within the LUP clade, exhibiting a close evolutionary relationship with reference LUPs from other species. In contrast, the remaining seven members (AktOSC2–AktOSC5 and AktOSC7–AktOSC9) partitioned into the broader triterpenoid synthase lineage but did not tightly cluster with any previously characterized reference OSCs. Notably, six of these members, with the exception of AktOSC5, formed an independent subclade, suggesting unique evolutionary trajectory of the OSC family within *A. trifoliata*.

### 3.4. Collinearity and Ka/Ks Analysis of OSC Family Members

**Figure 3:**
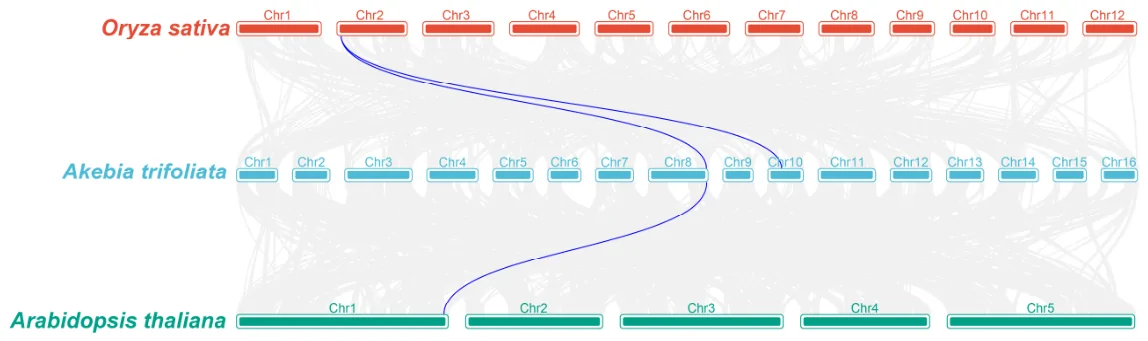
Collinearity analyses of the *OSC* genes between *A. trifoliata* and two reference species (*A. thaliana* and *O. sativa*). Blue lines represent collinearity among different *OSC* genes.

To elucidate the interspecific collinear relationship of the *OSC* genes, a comparative synteny analysis was performed between *A. trifoliata* and two representative plant genomes: the dicot *A. thaliana* and the monocot *O. sativa* (Figure 3). A total of three collinear *OSC* gene pairs were detected across these lineages. Specifically, two syntenic *OSC* gene pairs were identified between *A. trifoliata* and *O. sativa*, linking Chr2 of *O. sativa* to Chr8 and Chr10 of *A. trifoliata*. Additionally, one collinear gene pair was resolved between *A. trifoliata* (Chr8) and *A. thaliana* (Chr1). Notably, the *OSC* locus situated on Chr8 of *A. trifoliata* maintained highly conserved synteny with both monocot and dicot reference genomes, underscoring its ancestral origin and evolutionary stability across deeply divergent angiosperm lineages.

To assess the selective pressure among *AktOSC* pairs, Ka/Ks analysis was conducted for the nine members, generating 36 gene pairs in total (Table S2). All pairwise Ka/Ks ratios ranged from 0.15 to 0.35, indicating that the evaluated *AktOSC* gene pairs have predominantly evolved under purifying selection.

### 3.5. Cis-Acting Elements Composition of AktOSC Promoters

**Figure 4:**
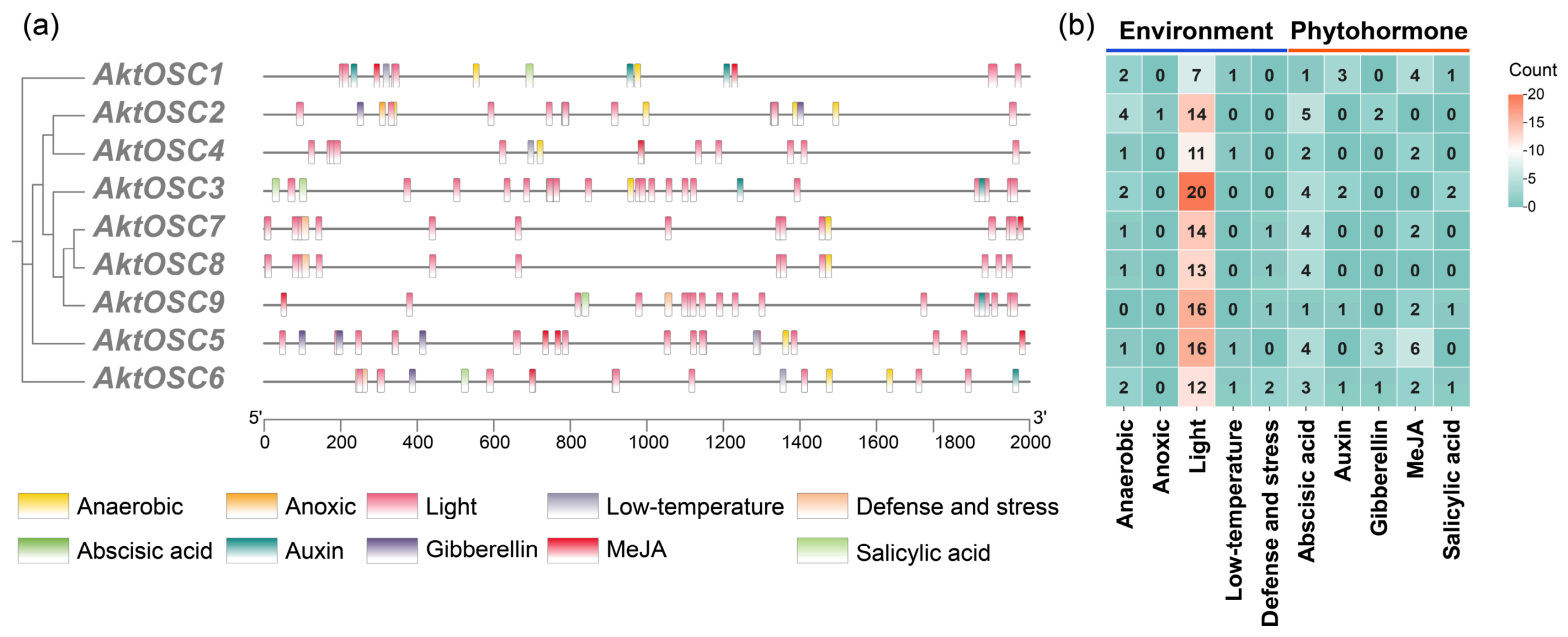
Prediction of cis-acting elements in the *AktOSC*s promoter. (**a**) The distribution of cis-acting elements in the 2000 bp upstream region of *AktOSC* genes. (**b**) The number of cis-acting elements of the two macro-categories in *AktOSC*s, indicated by different colors and numbers, respectively.

After removing basal promoter components (e.g., TATA-box and CAAT-box) and unannotated motifs from the raw predictions, a total of 211 functional cis-acting regulatory elements were identified within the 2000 bp upstream promoter regions of the nine *AktOSC* genes (Figure 4, Table S3). These elements were classified into two macro-categories encompassing 10 subcategories: environmental responsiveness (anaerobic-, anoxic-, light-, low-temperature-, and defense and stress-responsive elements) and phytohormone responsiveness (abscisic acid-, auxin-, gibberellin-, MeJA-, and salicylic acid-responsive elements). The abundance of cis-elements averaged 23.44 per gene, spanning from a minimum of 17 in *AktOSC4* to a maximum of 31 in *AktOSC5*. Within the two macro-categories, light-responsive and abscisic acid-responsive elements represented the most abundant subcategories, accounting for 58.3% and 13.3% of the total pool, respectively. Notably, these two functional groups were universally distributed across the promoters of all nine *AktOSC* genes. Conversely, anoxic-responsive and salicylic acid-responsive elements were the least represented in their respective macro-categories, with only one and five elements detected, respectively.

### 3.6. Expression Profiles of AktOSC Genes in Different Tissues and Stages

**Figure 5:**
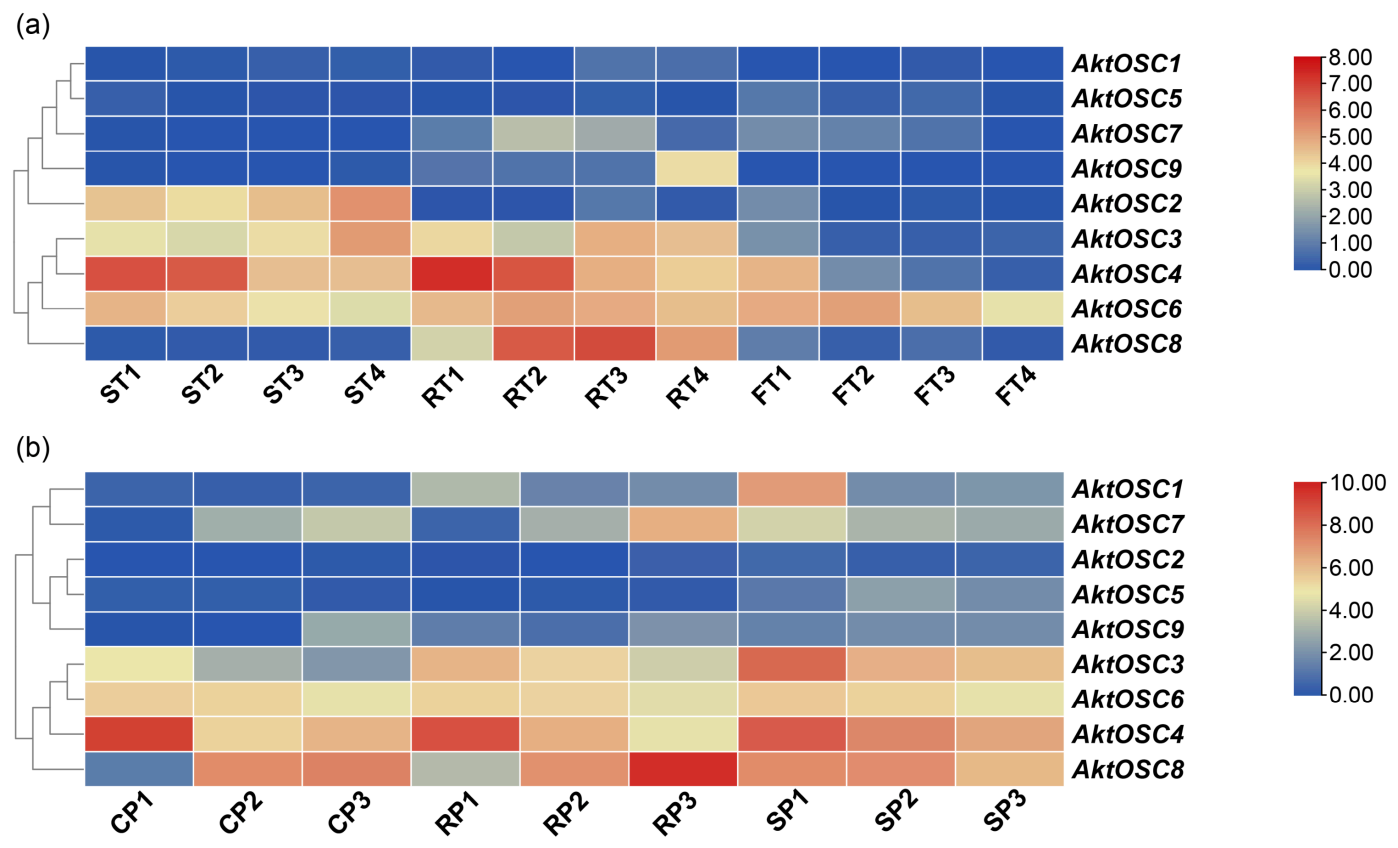
Expression profiles of *AktOSC*s in different stages. The degree of change from blue to red indicates the intensity of the expression level. (**a**) Expression profiles of *AktOSC*s in tissue- and development-related transcriptomes. ST, FT, RT and 1, 2, 3, 4 represent three different tissues (seed, flesh, and peel tissues) and four different developmental stages (immature, enlargement, coloring, and mature stages) of *A. trifoliata*, respectively. (**b**) Expression profiles of *AktOSC*s in disease-associated transcriptomes. CP, RP, SP and 1, 2, 3 represent three peel sample categories (control, disease-resistant, and disease-susceptible peel group) and sampling periods (May, June, and July) of *A. trifoliata*, respectively.

To elucidate the potential biological roles of *AktOSC* genes in *A. trifoliata* growth, development, and defense responses, their expression profiles were systematically evaluated using two transcriptomic datasets (Figure 5). Hierarchical clustering revealed pronounced spatial and temporal transcriptional divergence among the nine *AktOSC* genes during normal development. Specifically, *AktOSC2* exhibited strict seed-specific expression; its transcript abundance progressively increased during seed maturation and peaked at the mature stage (ST4), whereas it remained barely detectable in peel and flesh tissues. *AktOSC3* displayed moderate transcript accumulation restricted to the developmental sequences of both seeds and peels. Conversely, *AktOSC8* expression was uniquely restricted to the peel, showing a sharp preferential enrichment during the fruit enlargement (RT2) and coloring (RT3) stages. *AktOSC4* was heavily transcribed during early organogenesis, particularly in the immature and enlargement stage of seeds (ST1–ST2) and peels (RT1–RT2), before tapering off in later stages. In contrast to these highly tissue-specific patterns, *AktOSC6* maintained a relatively stable, moderate expression baseline across all examined tissues and stages, including the flesh. The remaining four members (*AktOSC1*, *AktOSC5*, *AktOSC7*, and *AktOSC9*) were transcriptionally quiescent, maintaining very low or undetectable expression levels throughout normal development.

Within the disease-associated transcriptome dataset, the *AktOSC* genes displayed distinct expression patterns across peel sample categories and sampling periods. Notably, *AktOSC8* showed a pronounced increase in expression in the disease-resistant peel group collected in July (RP3) and maintained relatively high expression in the disease-susceptible peel group collected from May to July (SP1–SP3). Intriguingly, *AktOSC1* and *AktOSC7*, which showed very low or undetectable expression in the fruit growth and development dataset, were detected at relatively high levels in some disease-associated peel samples. *AktOSC1* reached its highest observed expression level in SP1 (May), whereas *AktOSC7* showed relatively high expression in both RP3 and SP1. In contrast, *AktOSC3* and *AktOSC4* showed relatively high expression in the May samples of all three categories, followed by lower expression levels in June and July. Conversely, the remaining members (*AktOSC2*, *AktOSC5*, and *AktOSC9*) remained at very low or undetectable expression levels under all treatments.

## 4. Discussion

In plant triterpenoid biosynthesis, the cyclization of 2,3-oxidosqualene, catalyzed by OSC, is the initial committed step toward triterpenoid diversification, giving rise to more than 100 distinct triterpenoid skeletons [49]. Through heterologous expression in hosts such as yeast or tobacco, over 150 OSCs from more than 75 plant species have been functionally characterized to date [50]. Among these, bAS, CAS, and LUP constitute the most prevalent classes, accounting for 23.3%, 20.7%, and 14% of the total identified OSCs, respectively [50]. In the present study, nine *AktOSC* genes were identified in *A. trifoliata*. Phylogenetic analysis coupled with NCBI BlastP searches suggested that *AktOSC1* and *AktOSC6* may encode LUP and CAS (Figure 2, Table S4), respectively. In contrast, the remaining seven AktOSC proteins were positioned within the triterpene synthase-related branch, but they did not cluster closely with OSCs whose catalytic products have been experimentally characterized. Although their top BLASTP hits were annotated as bAS-like proteins, these sequence-based similarities are insufficient to establish their catalytic functions or product specificities. Given that minor modifications within the active site (such as a single amino acid substitution) can radically alter catalytic activity and yield diverse polycyclic triterpenoids [11], further biochemical verification is indispensable to conclusively determine the catalytic activities and product specificities of these AktOSC proteins.

### 4.1. Distinct Evolutionary Trajectories Shape the Functional Divergence of AktOSC Genes

Previous studies suggest that successive gene duplication and divergence events shaped the evolutionary trajectory of the plant OSC family, expanding OSC functions from ancestral roles in primary sterol biosynthesis in early-diverging plant lineages to increasingly diverse roles in specialized triterpenoid metabolism. Rather than resulting from a single ancestral radiation, this diversification appears to have involved repeated, lineage-specific innovations that generated distinct OSC repertoires across plant lineages [16,50]. Consistent with this evolutionary framework, most AktOSC members clustered within a distinct clade in the phylogenetic tree (Figure 2). Together with the identification of tandem, proximal, and dispersed duplicates (Table 1), this pattern suggests that multiple duplication modes contributed to the lineage-specific expansion and potential functional divergence of the AktOSC family. This finding agrees with the established consensus that numerous triterpene synthases evolved independently across diverse plant lineages [50]. Despite this lineage-specific expansion, the family-wide Ka/Ks analysis indicated that the *AktOSC* genes have experienced strong purifying selection (Table S2), suggesting that the duplicated members have remained under substantial evolutionary constraint, thereby preserving OSC functions important for sterol and triterpenoid biosynthesis in *A. trifoliata*.

Within this evolutionary framework, interspecific collinearity analysis was conducted among *A. trifoliata* (a basal eudicot), *O. sativa* (a monocot), and *A. thaliana* (a core eudicot) (Figure 3). Both *AktOSC1* and *AktOSC4* exhibited collinearity with the same OSC locus in *O. sativa*, indicating a possible one-to-many orthologous relationship. This pattern is consistent with their derivation from a common ancestral OSC followed by a lineage-specific duplication event in *A. trifoliata*. However, their subsequent evolutionary trajectories appear to have diverged. *AktOSC1* retained collinear relationships with OSC loci in both *O. sativa* and *A. thaliana*, suggesting that its surrounding genomic region has remained relatively conserved across the examined angiosperm lineages. When considered together with its phylogenetic placement, this conservation is compatible with its tentative annotation as a LUP. By contrast, *AktOSC4* retained detectable collinearity only with *O. sativa*, suggesting that its corresponding syntenic locus may have been lost or substantially reorganized along the lineage leading to *A. thaliana* through gene fractionation or localized genomic rearrangement. Similarly, the absence of detectable interspecific synteny for *AktOSC6* does not contradict its tentative annotation as a CAS, which is supported primarily by its phylogenetic placement and conserved sequence characteristics. Instead, this absence may reflect extensive restructuring of its surrounding genomic region over evolutionary time, whereas the conservation of its coding sequence is consistent with its putative role in essential sterol biosynthesis.

### 4.2. Conserved Motifs and Amino Acid Variations May Contribute to AktOSC Catalytic Diversity

The function of an enzyme is closely associated with its functional domains, and key amino acids within the active center frequently determine its catalytic activity. Therefore, understanding the relationship between these amino acids and enzyme structure and function is highly important. The DCTAE and QW motifs are conserved regions in OSC proteins [12,51]. The DCTAE motif is involved in substrate binding, and the aspartate (D) residue within this motif is responsible for substrate protonation to initiate the cyclization cascade [52,53]. Mutation of this specific residue can lead to a complete loss of catalytic capacity, whereas mutations at other positions within the motif may result in a drastic reduction in enzyme activity or diversification of the product profiles [4,11]. In this study, seven of the nine identified AktOSC proteins retained the canonical DCTAE motif (Figure S2), whereas AktOSC5 and AktOSC9 contained the variant sequences DCTGE and DGTAE, respectively. The QW motif features a consensus sequence of [K/R][G/A]X_2-3_[F/Y/W][L/V]X_3_QX_2-5_GXW [12], which is often simplified as QX_2-5_GXW [54], and its primary function is to stabilize carbocation intermediates during the cyclization cascade [55]. All AktOSC members contained conserved QW repeats conforming to the QX_2-5_GXW consensus. Furthermore, bASs generally possess a conserved MWCYCR motif, which is thought to participate in stabilizing complex cationic intermediates [10,56]. LUPs commonly contain an MLCYCR sequence at this position, whereas CASs and LASs frequently contain the corresponding MWCHCR sequence [10]. Consistent with previous findings, AktOSC1 (putatively inferred as a LUP) and AktOSC6 (putatively inferred as a CAS) harbored the MLCYCR and MWCHCR motifs, respectively. Most other members predicted to be bAS featured the conserved MWCYCR motif, except for AktOSC3 (MWIYCR) and AktOSC7 (MWCHCR). In conclusion, minor variations within the active site can substantially influence enzyme activity and product profiles, representing one of the key mechanisms driving the diversification of plant triterpenoid metabolism. Nevertheless, the functional predictions for these AktOSC proteins require subsequent molecular and biochemical experiments to definitively confirm their enzymatic identities.

### 4.3. Promoter Cis-Elements and Developmental Expression Patterns Suggest Spatiotemporal Regulation of AktOSC Genes

Numerous studies have demonstrated that different members of the plant OSC family often exhibit distinct tissue-specific expression patterns, indicating that they fulfill diverse biological functions in growth and development, chemical defense, and environmental adaptation [4,57,58]. In the present study, the promoter regions of the *AktOSC* genes contained diverse predicted cis-acting elements associated with environmental and hormonal responses, providing sequence-based clues to their potential differential regulation during fruit development. Among these, light-responsive elements are widely enriched across all *AktOSC* promoters and represent the most abundant cis-element subcategories for each member (up to 20), implying that light signaling may be a key upstream environmental factor driving triterpenoid accumulation in *A. trifoliata*. For instance, *AktOSC8*, which was almost exclusively expressed in peel, contained 13 predicted light-responsive elements and exhibited relatively high transcript abundance during the immature and enlargement stages (RT1–RT2). This expression pattern broadly corresponds to the previously reported accumulation of triterpenoids in *A. trifoliata* peel during early fruit development [59], supporting the prioritization of *AktOSC8* as a candidate potentially involved in peel triterpenoid biosynthesis. In contrast, *AktOSC2*, which was almost exclusively expressed in seeds and reached its highest transcript abundance at the mature stage (ST4), contained five predicted abscisic acid-responsive elements in its promoter. Given the established role of abscisic acid in seed maturation and dormancy, these observations suggest that *AktOSC2* may be associated with triterpenoid biosynthesis during seed development. Additionally, *AktOSC3* and *AktOSC4*, which were predominantly expressed in seeds and peels, exhibited opposing temporal expression trends. These patterns suggest possible temporal partitioning among *AktOSC* members during seed and peel development, although their precise metabolic roles remain to be determined.

### 4.4. AktOSC Genes Exhibit Distinct Expression Patterns in Disease-Associated Peel Samples

Within the disease-associated transcriptome dataset, the *AktOSC* genes also exhibited distinct expression patterns across peel samples of various categories and at different sampling periods, suggesting their potential involvement in the plant’s defense mechanisms. For example, *AktOSC8* exhibited an increasing expression trend from May to July in the control and disease-resistant peel samples, whereas it showed a decreasing trend across the same sampling periods in the disease-susceptible peel samples, suggesting that its expression may be associated with variation in disease susceptibility among the sampled materials. Given the established defensive roles of some plant triterpenoids [60], *AktOSC8* may represent a candidate for investigating the possible relationship between triterpenoid biosynthesis and disease-associated physiological responses. Interestingly, although *AktOSC1* and *AktOSC7* showed very low transcript abundance in the fruit developmental dataset, their transcripts were detected at relatively high levels in some disease-associated peel samples. These qualitative differences suggest possible context-dependent regulation.

### 4.5. Integrated Sequence and Expression Analyses Prioritize Candidate AktOSC Genes for Triterpenoid Biosynthesis

Integration of the conserved-motif, phylogenetic, and expression analyses allowed the *AktOSC* genes to be prioritized for subsequent functional characterization.

The DCTAE motif is directly involved in substrate binding and initiation of the 2,3-oxidosqualene cyclization reaction, making sequence conservation within this region particularly important for OSC catalytic function. AktOSC5 and AktOSC9 contained the noncanonical DCTGE and DGTAE sequences, respectively, and these substitutions may substantially affect their catalytic activities [11]. Together with their very low transcript abundance within both the fruit developmental and disease-associated datasets, these results substantially reduce the priority of AktOSC5 and AktOSC9 as candidates for the predominant triterpenoid biosynthetic pathways.

Variations in the MWCYCR-related region may influence OSC product specificity [10]. The phylogenetic positions and characteristic MLCYCR and MWCHCR sequences of AktOSC1 and AktOSC6 support their tentative annotations as LUP and CAS, respectively. Therefore, these proteins are less likely to encode the β-amyrin synthases primarily responsible for forming the oleanane-type scaffolds that predominate among the bioactive triterpenoids of *A. trifoliata* [18]. The relatively constitutive expression of *AktOSC6* across the examined tissues and developmental stages is also consistent with its predicted role in primary sterol biosynthesis. By contrast, the catalytic functions of AktOSC3 and AktOSC7 remain uncertain because their variant MWIYCR and MWCHCR sequences alone are insufficient to determine product specificity. Moreover, although *AktOSC1* and *AktOSC7* showed very low transcript abundance in the fruit developmental dataset, their transcripts were detected at appreciable levels in some disease-associated peel libraries. These qualitative differences indicate that they may be associated with plant defense.

AktOSC2, AktOSC4, and AktOSC8 retained the canonical DCTAE and MWCYCR motifs and exhibited relatively high, tissue-associated expression. *AktOSC2* was predominantly expressed in seeds, *AktOSC8* was expressed in the peel, and *AktOSC4* was expressed in both tissues. Together, these sequence and expression characteristics prioritize these three members as candidates potentially involved in triterpenoid biosynthesis. In particular, the developmental expression profile of *AktOSC8* broadly corresponded to the previously reported accumulation pattern of triterpenoids in fruit peels [59], making it the highest-priority candidate. Elucidating the precise relationships between these candidate genes (particularly *AktOSC8*) and triterpenoid accumulation via experimental validation is our next objective.

## 5. Conclusions

In this study, nine *AktOSC* genes were identified from the reference genome of *A. trifoliata*, and their physicochemical and structural characteristics were analyzed. Phylogenetic analysis indicated that most AktOSC proteins formed independent evolutionary subclades, suggesting a unique evolutionary trajectory of this family in *A. trifoliata*. Synteny and selective pressure analyses revealed that multiple duplication modes, including tandem, proximal, and dispersed duplications, collectively contributed to the expansion of the AktOSC family, and all members were subjected to strong purifying selection. Combined analysis of promoter cis-acting elements and expression patterns demonstrated that the *AktOSC* genes exhibited distinct spatiotemporal transcriptional divergence during normal fruit growth and development. Among these genes, *AktOSC8* exhibited relatively high, peel-enriched expression and contained numerous predicted light-responsive cis-elements, suggesting its potential association with light-related regulation and triterpenoid accumulation in the peel. In the disease-associated transcriptome dataset, the *AktOSC* genes displayed distinct expression patterns among peel samples from different disease-susceptibility groups, and *AktOSC8* showed contrasting temporal trends between disease-resistant and disease-susceptible samples. These observations identify *AktOSC8* as a candidate for further investigation of the possible relationship between triterpenoid biosynthesis and disease-associated physiological responses. In summary, this study characterized the basic features, evolutionary relationships, predicted regulatory elements, and spatiotemporal and disease-associated expression patterns of the *AktOSC* gene family in *A. trifoliata*, providing a theoretical foundation for further deciphering the regulatory mechanisms of triterpenoid biosynthesis and facilitating molecular breeding for disease resistance in *A. trifoliata*. Future studies could focus on utilizing molecular biology techniques to perform in vivo and in vitro functional characterization of key members (such as *AktOSC8*) and functional validation of cis-acting elements, thereby fully elucidating their molecular regulatory networks.

## Figures and Tables

**Table 1:** Characteristics of the identified *AktOSC* genes and putative AktOSC proteins.

Gene ID	Gene Name	Chromosome	Location	Gene Length (bp)	CDS Length (bp)	Exon No.	Duplication Type	Putative Protein						
								Protein Length (AA)	Molecular Weight (kDa)	Theoretical pI	Instability Index	Aliphatic Index	GRAVY	Subcellular Localization Prediction
EVM0000200.1	*AktOSC1*	Chr8	50795612–50810804	15,192	2289	19	Proximal	762	87.49	6.03	50.13	77.97	−0.336	Endoplasmic reticulum
EVM0015974.3	*AktOSC2*	Chr8	50892089–50902659	10,570	2310	18	Tandem	769	88.26	6.27	46.3	76.85	−0.333	Endoplasmic reticulum
EVM0000305.3	*AktOSC3*	Chr8	50909635–50934191	24,556	2304	19	Tandem	767	88.24	5.97	45.65	78.07	−0.309	Endoplasmic reticulum
EVM0006717.3	*AktOSC4*	Chr10	12148994–12160850	11,856	2301	19	Dispersed	766	87.83	5.95	46.83	79.57	−0.269	Endoplasmic reticulum
EVM0017931.1	*AktOSC5*	Chr11	50177094–50189525	12,431	2289	18	Proximal	762	87.52	5.86	47.54	80.66	−0.358	Endoplasmic reticulum
EVM0012719.1	*AktOSC6*	Chr15	3284306–3298638	14,332	2121	20	Dispersed	706	80.31	5.99	36.6	81.94	−0.298	Endoplasmic reticulum
EVM0024033.1	*AktOSC7*	Contig00522	2624–27262	24,638	2187	17	Tandem	728	83.83	6.51	48.37	78.79	−0.372	Endoplasmic reticulum
EVM0014306.1	*AktOSC8*	Contig00522	36975–50231	13,256	2268	18	Tandem	755	87.15	6.41	43.66	80.24	−0.313	Endoplasmic reticulum
EVM0012151.1	*AktOSC9*	Contig00577	25444–38997	13,553	2310	18	Proximal	769	88.32	6.17	43.98	81.29	−0.298	Endoplasmic reticulum

**Table 2:** Information on the conserved motifs of AktOSC proteins.

Motif	Width	Best Possible Match	OSC Domain
Motif1	75	LGVYEWSGCNPMPPEFWLLPSFLPMHPGKMWCYCRLTYMPMSYLYGKRFVGPITDLILSLREELYIQPYHEIBWN	MWCYCR
Motif2	75	VRFFSAMQTSDGHWAAEIGGPLFFMPPLVFVLYITGMLDTIFSAEHKKEILRYMYYHQNEDGGWGFHIEGHSTMF	QX_2-5_GXW
Motif3	75	SQMWDTSFAVQALIASNLHDEIGHTLKKGHDYIKASQVKDNPSGDFKSMYRHISKGAWTFSDQDHGWQVSDCTAE	DCTAE
Motif4	75	TRWPFNKYREKALQVTMKHIHYEDENSRYMTIGAVEKVLCMLACWVEDPNSBAFKKHLARIPDYLWVAEDGMRMQ	
Motif5	75	NLVQTAWALMGLIYAGQAEIDPTPLHRAVKVLINNQMENGDFPQQEITGVFMKNCMLHYAAYRNIFPLWALAEYR	
Motif6	66	QMPDGSWYGNWGICFIYGTWFALRGLSAAGKNYYNSPTVRKATEFLLSTQQPSGGWGESYRSCPEK	QX_2-5_GXW
Motif7	61	AKESVSGPYSPWLFSTNNFIGRQTWEFDPDAGTPEERAEVEKAREEFRRNRFQVKPCGDVL	
Motif8	75	SLQSKNGGLAAWEPVRAHEWLEVLNPTEFFZDIVVEHEYVECTASAIZALVCFMKLYPGHRKKEIENFIAKAIRY	

## Data Availability

The original contributions presented in this study are included in the article/Supplementary Materials. Further inquiries can be directed to the corresponding author.

## Supplementary Materials

The following supporting information can be downloaded at https://www.mdpi.com/article/10.3390/cimb48080795/s1.

## Abbreviations

The following abbreviations are used in this manuscript:

OSC	Oxidosqualene cyclase
C-B-C	Chair–boat–chair
C-C-C	Chair–chair–chair
CAS	Cycloartenol synthase
LAS	Lanosterol synthase
LUP	Lupeol synthase
DDS	Dammarenediol synthase
bAS	β-amyrin synthase
TCM	Traditional Chinese medicine
HMM	Hidden Markov model
FPKM	Fragments per kilobase of transcript per million mapped fragments
ML	Maximum likelihood
